# Surgery simulation teaching based on real reconstruction aid versus traditional surgical live teaching in the acquisition of an adult total hip arthroplasty surgical technique for developmental dysplasia of the hip: a randomized comparative study

**DOI:** 10.1186/s12909-020-02135-z

**Published:** 2020-07-20

**Authors:** Chenggong Wang, Yang Ouyang, Hua Liu, Can Xu, Han Xiao, Yihe Hu, Yusheng Li, Da Zhong

**Affiliations:** 1 Office of teaching affairs, Xiangya Hospital, Central South University, Changsha, Hunan China; 2 Department of Orthopedics, Xiangya Hospital, Central South University, Changsha, Hunan China; 3 Department of Sports Medicine, Xiangya Hospital, Central South University, Changsha, Hunan China

**Keywords:** Surgical technique, Continuing education training, Joint surgery, Surgery simulation teaching, Real reconstruction model aid

## Abstract

**Background:**

A simulation and model (SM) teaching aid using 3D printing was developed to improve a training course for total hip arthroplasty of adult developmental dysplasia of the hip (adult DDH-THA). We named this new method Surgery Simulation Teaching based on a Real Reconstruction Aid (RRA-SST). A prospective randomized comparison was performed with the traditional surgical live teaching method to evaluate the training effectiveness of RRA-SST for adult DDH-THA.

**Methods:**

Twenty-six trainees, who were already practicing but were not experienced, participated in the study. We randomly divided the trainees into two groups: Group A (*n* = 13) received RRA-SST and group B (*n* = 13) received traditional surgical live teaching. A surgery simulation test and a questionnaire were used for evaluation. Next, each group received training with the other teaching method, and then the test and questionnaire were used again for evaluation.

**Results:**

After the first test, the RRA-SST method was shown to produce better results than the traditional surgical live teaching method. After the second test, the results showed the training effect in both groups reached the same level, which was level as Group A RRA-SST results. Analysis of the questionnaire results showed that the training effect of RRA-SST was higher than that of traditional surgical live teaching, from multiple perspectives.

**Conclusions:**

The use of RRA-SST improved participant performance according to simulation assessment. RRA-SST can be helpful for trainees who are already practicing but not experienced when developing proficiency in adult DDH-THA surgical techniques.

## Background

Most clinicians in China, including senior doctors, lack a Doctor of Medicine (M.D.) degree [1]. In addition, resident training and the medical education system are in the early stages of development in this country [2, 3]. Therefore, continuing education of Chinese clinicians who work in basic hospitals, or in hospitals that are not highly rated, is critical [4, 5]. In China, hospitals rated as regional medical centers at or above the provincial level are considered “top” hospitals, while the rest are regarded non-top hospitals. In recent years, with deepening of China’s medical reform, the equipment used by basic and non-top hospitals has improved, and more patients are admitted to these hospitals for treatment [6, 7]. As a result, top hospitals or regional medical centers of China are expected to assume increasing responsibility for continuing education and training of basic hospital doctors through short-term surgical technique training programs.

Developmental dysplasia of the hip (DDH) is a congenital acetabular malformation [8]. The prevalence of DDH varies from 1.6 to 28.5 cases per 1000 live births depending on the definition and the population being studied [9, 10]. Up to 94% of adults with untreated congenital dislocation of the hip will have moderate to severe osteoarthritis by the second decade [11]. DDH prevalence is more than 2 % in China [12]. In the Norwegian Arthroplasty Register, DDH was implicated in 9% of all primary total hip arthroplasties (THAs) and almost one third of THA cases occurred in people under 65 years old [13]. In China, DDH is the reason for up to 9% of THAs and up to 29% of the THAs occurred in people aged 60 years and younger [14]. THA is usually the preferred treatment for advanced and severe adult DDH [15].

**Fig. 1:**
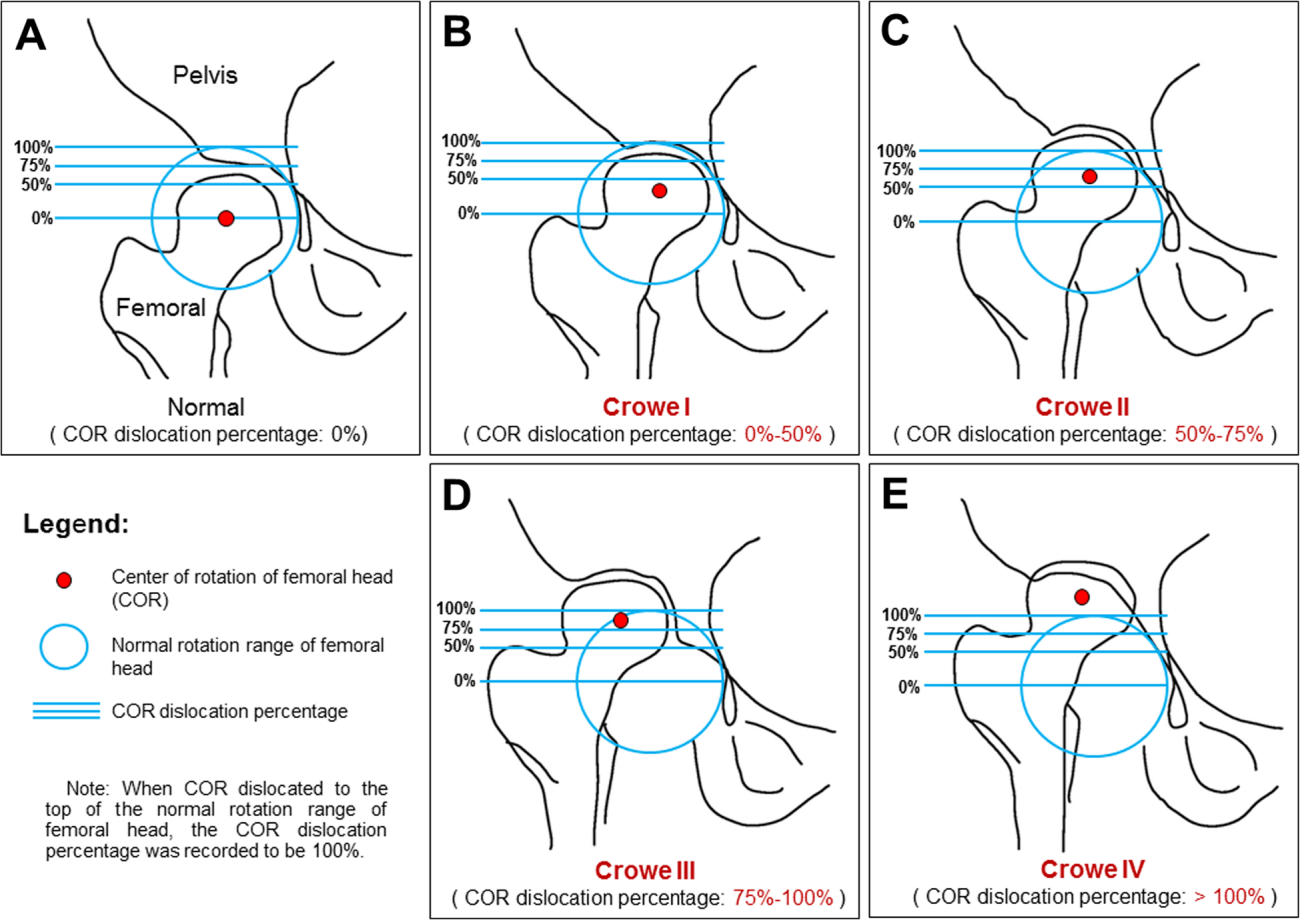
Schematic diagram of normal hip and Crowe’s classifications for adult developmental dysplasia of the hip (DDH); red dots represent the center of rotation of the femoral head; multi-colored area represents the degree of subluxation of the femoral head: 0, 50, 75, and 100% from bottom to top. **a** Front view of the normal hip joint, the femoral head is not dislocated, and has a normal structure; **b** Front view of Crowe’s classification I DDH, subluxation of the femoral head less than 50%; **c** Front view of Crowe’s classification II DDH, subluxation of the femoral head 50–75%; **d** Front view of Crowe’s classification III DDH, subluxation of the femoral head 75–100%; **e** Front view of Crowe’s classification IV DDH, subluxation of the femoral head more than 100%

At present, most clinics use Crowe’s classification system, which was proposed by Crowe et al. in 1979 [16], to evaluate adult DDH. The authors differentiated four classes of adult DDH. Group I was defined as less than 50% subluxation of the femoral head, group II as 50–75% subluxation of the femoral head, and group III as 75–100% subluxation of the femoral head. Group IV was assigned to a proximal migration of more than 100% of the femoral head (Fig. 1). Crowe’s classification system has high practicality and reliability and it is commonly used to evaluate the degree of hip anatomy abnormality [17]. The success of total hip arthroplasty depends on the degree of anatomical abnormality and whether the anatomical structure can be reconstructed perfectly [18]. The “rotation center”, “anteversion angle”, and “inclination angle” are important reference indicators for evaluating whether the acetabulum can be reconstructed perfectly [19]. Wrong positioning of the rotation center and the wrong size anteversion and abduction angles of the hip have been shown to increase the risks of postoperative complications; e.g., abductor muscle weakness, dislocation [20], and aseptic loosening [21].

In China, the training method for adult DDH-THA, especially for clinicians from basic hospitals or non-top hospitals, is highly dependent on continuing education. With the deepening of medical reform in China, an increasing number of adult DDH-THA patients will be admitted to basic hospitals for surgical treatment [22]. However, currently, basic hospital doctors in most parts of China have not mastered this technique. Moreover, the number of adult DDH-THAs completed by basic hospitals is low, and most of the basic hospitals are not qualified for teaching this technique. Therefore, there is a huge demand for teaching and training clinicians to perform adult DDH-THAs in China.

Xiangya Hospital Central South University, where our team is located, is one of the regional medical centers in China. The hospital covering Hunan province and five of the neighboring provinces is very large, and the number of adult DDH-THAs performed in the hospital is very high. To carry out adult continuing education for DDH-THA better and accelerate the learning curve for the operating technique, our team has held 10 short-term training sessions on the adult DDH-THA surgical technique since October 2017 to date. However, the adult DDH-THA training used the traditional teaching method, which combines theoretical discussions with live surgical observations using broadcasting equipment. A problem was revealed, however, that needed an immediate solution; that is, the traditional teaching method lacked hands-on training. The reason is cadaver specimens of adult DDH are very rare in China and it is difficult to carry out adult DDH-THA simulated surgery using cadaver specimens. Many trainees suggested that some hands-on training would improve the teaching method. Therefore, our teaching team aimed to improve the training method regarding several aspects to achieve better training results for adult DDH-THA.

Six years ago, our hospital created the Digital Research Institute of Orthopedics and introduced the use of 3D printing machines. Five years ago, the institute mastered digital modeling of bone and printing technology for bone models. Four years ago, simulations and models (SM) [23] were applied in our hospital orthopedic clinical training program and, in recent years, this program was generalized to other clinical professional teaching programs. Three years ago, a number of orthopedic surgeons in our hospital successively used preoperative surgery simulation on a computer and 3D printed models when formulating the preoperative plan for adult DDH-THA. In the past 3 years, technical improvement of the preoperative plan received good feedback and has been promoted further. At present, almost all orthopedic surgeons use the preoperative computer surgery simulation and 3D printed model simulation to carry out the preoperative surgical program design for difficult orthopedic surgeries, such as adult DDH-THA.

In an attempt to further improve the curriculum, and based on the above favorable conditions, our team introduced SM into the DDH-THA surgery teaching programs 2 years ago. We developed an artificial model that can be used to fully restore the patient’s bone and soft tissue conditions within several attempts. The bone part of this model was prepared using a 3D printing method and the soft tissue was made using silica gel. After two rounds of revisions in the Clinical Medicine Teaching Department of our hospital, the SM was formally applied to the continuing education for DDH-THA. Positive feedback from trainees and trainers was received after applying this new teaching aid. Subsequently, we adjusted and optimized this new teaching aid many times and gradually formed a mature and reliable SM and a set of standardized teaching methods. Moreover, we named this new method Surgery Simulation Teaching Based on Real Reconstruction Aid (RRA-SST). During the last three semesters of medical education, the SM were applied to the DDH-THA surgery teaching program six times.

There have been no comparative studies between this type of adult DDH-THA simulation training program and traditional training methods to the best of our knowledge. Therefore, we designed this prospective randomized comparison study to evaluate the teaching effectiveness of RRA-SST applied to adult DDH-THA surgical training. In view of the great demand for clinical continuing education in China, the development of this type of training program may have a positive influence on additional clinical specialties and surgical techniques.

## Methods

### Participants

This study was approved by the ethics committee of the Xiangya Hospital of Centre South University [IRB (C) No. 201903055]. Participants were recruited from trainees of the senior class at Xiangya Hospital of Centre South University in early October 2019. This senior class was participating in an advanced course that was approved by the Continuing Education Committee of the Chinese Medical Association. The advanced course is open to all Chinese surgeons who have obtained a doctor’s license and have at least 2 years of experience in THA surgery. Usually, these young surgeons will begin performing adult DDH-THAs after receiving traditional training and passing the completion test for this advanced course. The trainees participating in this study did not know the design of this research project before agreeing to participate. They also did not know the surgery simulation link for model teaching AIDS was added to the training course. However, after arriving at the training site, the trainees were informed of the purpose and protocol for this research project. On basis of the relevant research [24, 25], a minimum of 12 participants were required for each group according to a power analysis (alpha = 0.05, beta = 0.2).

Thirty-nine trainees were participating in the training course and 36 of them were chosen to participate in the study. To reduce confounding factors, we removed 10 trainees for the following reasons: they had less than a bachelor’s degree (*n* = 2), had less than a bachelor’s degree and rank below attending doctor (*n* = 1), had a rank below attending doctor (*n* = 2), had a title that outranked the associate chief surgeon (*n* = 1), were surgeons already performing adult DDH-THAs (*n* = 3), and not planning to engage in DDH-THA in the future (*n* = 1). Finally, 26 participants were enrolled and a random number generator was used to divide the participants into Group A (*n* = 13) and Group B (*n* = 13; Table 1). Informed consent was obtained from all individual participants included in the study.

**Table 1:** Demographic of the participants

	group A	group B	P
Age (years)	33.38 ± 3.88	33.77 ± 4.25	0.703
Gender (male/female)	M/F (13/0)	M/F (13/0)	1.000
THA experience (surgical amount)	3.00 ± 1.47	4.08 ± 2.53	0.200

P: *P* values (Independent-samples t-test)

### Teaching aids preparation

Two senior chief surgeons who had completed more than 50 adult DDH-THAs as the main surgeon selected 12 typical cases. Then a random number generator was used to randomly divide the typical cases into group-M1, group-M2, and group-M3. Group-M1 and group-M2 were set as the trainee-operation group and group-M3 was set as the teaching group. Each group contained four cases and each case corresponded to one of the Crowe’s classification types. Each case was numbered as Crowe’s classification x-y, (x represented type Crowe’s classification I to type Crowe’s classification IV, y represented Group A to Group C). To reduce the interference factors, the two senior chief surgeons selected typical cases to ensure that the bone and joint capsule conditions of the cases were different and also ensured the surgical procedures were not different in each Crowe’s classification subgroup.

**Fig. 2:**
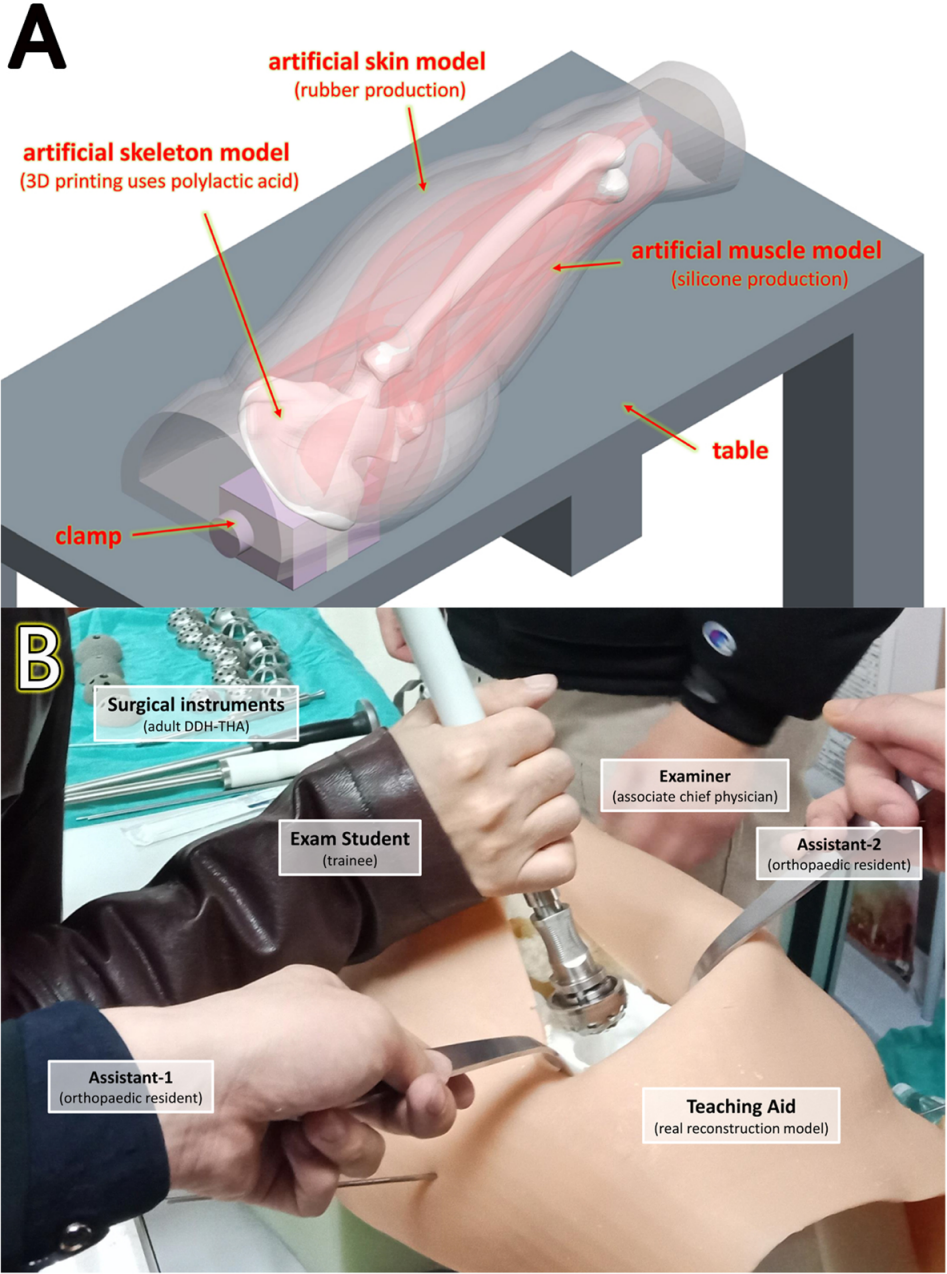
**a** Schematic diagram of the real reconstruction model aid: a full-size hemipelvis model using 3D printing, simulated soft tissue was applied to cover the model; **b** The trainee was tested using simulation surgery, all surgeries used the same kind of surgical instruments, and there were two residents assigned to assist the trainee with one examiner giving the score

We used Mimics 19.0 software (Materialise, Leuven, Belgium) to reconstruct the pelvic area. The pelvic position was standardized with reference to the anterior pelvic plane. A full-sized hemipelvis model was printed using polylactic acid (PLA) connected with a cube base that could be fixed on the surgery table. This kind of material could be ground in a similar manner to bone. When the base was fixed on the surgery table, the model was in a standard lateral position and it could be prepared using a posterior approach, which was the most common approach used by all participants. A simulated soft tissue was applied to cover the model (Fig. 2a). To reduce interference factors, we adjusted left and right positions 1:1 during reconstruction of the patient’s pelvis to ensure the sides of group-M1 and group-M2 in each Crowe’s classification subtype were different.

### Surgery simulation and model measurement

All surgery simulations were completed in one of seven classrooms, and the hardware and lighting conditions of each classroom were consistent. All the surgeries used the same kind of surgical instruments and cup products (Deputy, Johnson & Johnson, USA, seven sets of use, two sets of standby). The trainees were allowed to view the X-rays and CT scans of the model 5 min before the surgery. The operation time was recorded from the time of skin cutting to the time after the cup was installed. The pelvis and the cup were taken out as a whole. Unnecessary anatomical injuries were judged first, and then the cup orientation was examined.

The details were as follows: The postoperative hip with a prothesis was scanned immediately with a 3D intelligent scanning instrument (OKIO-5 M-100, Beijing Tenyoun 3d Technology Co., Ltd). We exported the scanning data (format: STL) to reconstruct the digital virtual hip postoperatively. We recorded the postoperative cup position and direction data, including the ideal rotator center discrepancy (IRCD), inclination, and anteversion. The IRCD was used to evaluate the cup position, and the inclination and anteversion were used to evaluate the cup direction. The measurement error of the software was 0.005 mm.

In every classroom, there were two residents (a total of 14) assigned to help the students through auxiliary work such as grabbing hand instruments and maintaining retractors, and there was one examiner (associate chief surgeon, a total of seven) giving a score for the surgery simulation test (Fig. 2b). The details of the steps and errors were recorded (Table 2). The differences in cup sizes between the ideal and actual were analyzed and recorded as the ideal cup size discrepancy (ICSD). To reduce interference, the seven examiners and 14 residents were uniformly trained and evaluated by the two senior chief surgeons who selected the cases to be included in the study. All simulation model reconstruction in this study was completed by one technician under the guidance of the two senior chief surgeons who selected the cases.

**Table 2:** Score rules of the real reconstruction model surgery simulation test

**SURGERY SCORE RULES (score range: 1–100)**	
**A. Rules of plus** (score limit: 100):	
1. Is the incision located correctly? (full score: 10)	
2. Is the incision directed correctly? (full score: 5)	
3. Is the incision length appropriate? (full score: 5)	
4. The process that found soft tissue gap of the approach is accurate? (full score: 5)	
5. The exposure of the joint is comfortable? (full score: 10)	
6. The process that determine true acetabulum is quick? (full score: 10)	
7. The grinding of the acetabulum is smooth? (full score: 5)	
8. Is the acetabulum reconstructed qualified? (full score: 20)	
9. Is the choice of cup model appropriate? (full score: 10)	
10. Is the installation of cup qualified? (full score: 20)	
**B. Rules of deduction** (range of score deducted: 1–50):	
1. If the acetabulum is worn. (1–20 points, depending on the situation)	
2. Operation is biased toward posterior important nerves area. (1–30 points, depending on the situation)	

### Design

**Fig. 3:**
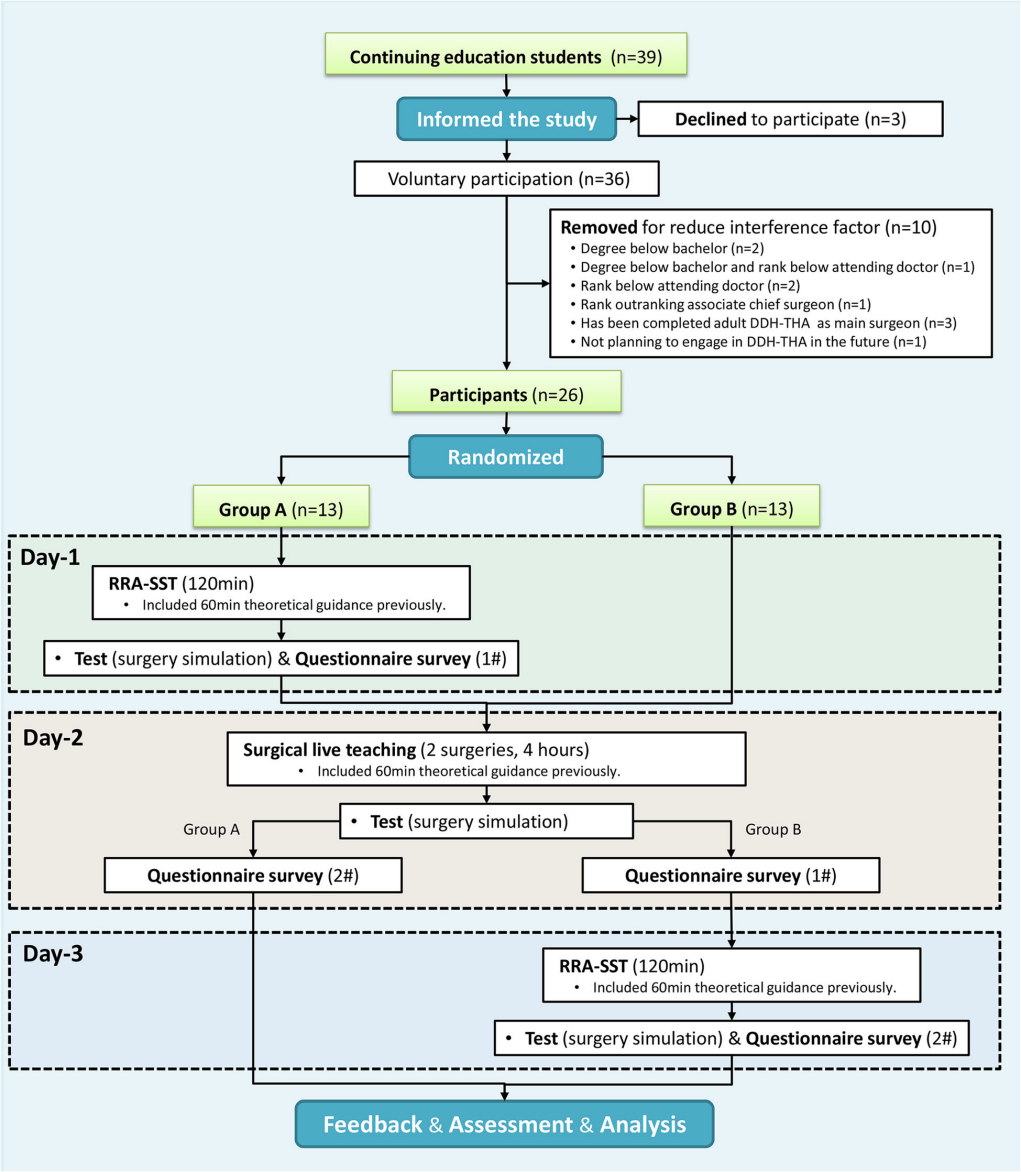
Design methodology for randomized comparison research

To reduce the interference factors, all theoretical and surgical teaching sessions were conducted by two senior chief surgeons who had teaching experience of at least 3 years or 90 class-hours. In addition, the models used in the two surgery simulation tests were randomly selected from groups-M1 and group-M2. The steps were as follows (Fig. 3, Table 3):

1. On the first day, all Group A trainees accepted training using RRA-SST. They received theoretical guidance first (60 min) and then received simulated surgery training using the real reconstruction rid model (120 min). In the simulation surgery segment, the teaching model adopted all four Crowe’s classification subtypes of group-M3. Next, all Group A trainees completed the surgery simulation test using the real reconstruction rid model independently (group-M1 or group-M2). No other trainees were allowed to watch the operation and all Group A trainees completed questionnaire #1 after the test. Relevant measurements and analyses were performed during or after the test.
2. On the second day, all Group A and Group B trainees received live surgical live training together. All participants received theoretical guidance first (60 min) and then watched the live surgery (typical case 1#, Crowe’s classification III, 118 min; typical case 2#, Crowe’s classification IV, 139 min). Next, all Group A and Group B trainees completed the surgery simulation test using the real reconstruction rid model independently (group-M1 or group-M2). No other trainees were allowed to watch the operation. After the test, all group A trainees completed questionnaire #2 and all Group B trainees completed questionnaire #1. Relevant measurements and analyses were performed during or after the test.
3. On the third day, all Group B trainees received training using RRA-SST. The specific steps were the same as in step (1) above.

**Table 3:** Issues Assessed in the Questionnaire

**Questionnaire 1#:**	
**A. Demographic and general informations:**	
1. Gender (single choice: 1.male; 2.female)	
2. Age (years)	
3. I have previous experience of surgery simulation by using orthopedic model (single choice: 1.yes; 2.no)	
**B. Questions*(single choice: 1.strongly disagree; 2.disagree; 3.equivocal; 4.agree; 5.strongly agree; 6.do not know):***	
1. This theoretical guidance segment is very helpful for me to master the surgical technique.	
2. The second teaching segment^a^ of this training is very helpful for me to master the surgical technique.	
3. This teaching can guide me to complete the surgery simulation well.	
4. This teaching can guide me to complete real adult DDH-THA well in the future.	
5. From now on, I have self-confidence enough to perform a real adult DDH-THA independently.	
**Questionnaire 2#:**	
**A. Questions*(single choice: 1.strongly disagree; 2.disagree; 3.equivocal; 4.agree; 5.strongly agree; 6.do not know):***	
1. From now on, I have self-confidence enough to perform a real adult DDH-THA independently.	
2. This new model reproduced adult DDH in a realistic way.	
3. Operate on this new model reproduced adult DDH-THA in a realistic way.	
4. For surgical technique training of adult DDH-THA, the teaching effect of RRA-SST is better than surgical live teaching.	
5. RRA-SST should be included in the course in the future.	
6. For surgical technique training of adult DDH-THA, the teaching effect of RRA-SST is better than other traditional teaching methods.	
7. For surgical technique training of adult DDH-THA, it is better that conduct RRA-SST first and then conduct live surgical teaching.	
8. Overall, I think RRA-SST is very useful in orthopedic surgery teaching.	
**B. Questions*(multiple choice):***	
1. Which Crowe types of adult DDH-THA surgical training needs RRA-SST? (1. Crowe I; 2. Crowe II; 3.Crowe III; 4.Crowe IV)	

^a^Group A: RRA-SST; Group B: Surgical Live Teaching

### Statistical analysis

All of the collected data were categories. An independent samples t-test was used to assess the differences between Group A and Group B at the same stage. The significance level was set at *p* < 0.05. All statistical analyses were performed using SPSS version 20.0 (IBM Corp., Armonk, NY, USA).

## Results

### Participants

Finally, 26 participants were enrolled. The demographic data showed all participants were male, the average age was 32.00 ± 4.00, all of them were attending doctors or associate chief surgeons, and all of them had a degree at or above the bachelor’s level. All participants had never completed an adult DDH-THA as the main surgeon. According to the survey, however, every participant completed 4.00 ± 2.10 simple adult THAs. Furthermore, the average age of Group A was 33.38 ± 3.88 and the average age of group B was 33.77 ± 4.25, with no statistically significant difference (*P* = 0.703). The average amount of experience with THA surgeries in Group A was 3.00 ± 1.47, and in Group B it was 4.08 ± 2.53, with no statistically significant difference (*P* = 0.200; Table 1). There were two participants in each group that had previous experience with surgery simulation using an orthopedic model.

### Surgical skills assessment

Before the first surgery simulation test, Group A only received training with RRA-SST, while Group B received training with live surgeries. Before the second test, both Groups A and B received training with the two teaching methods.

**Fig. 4:**
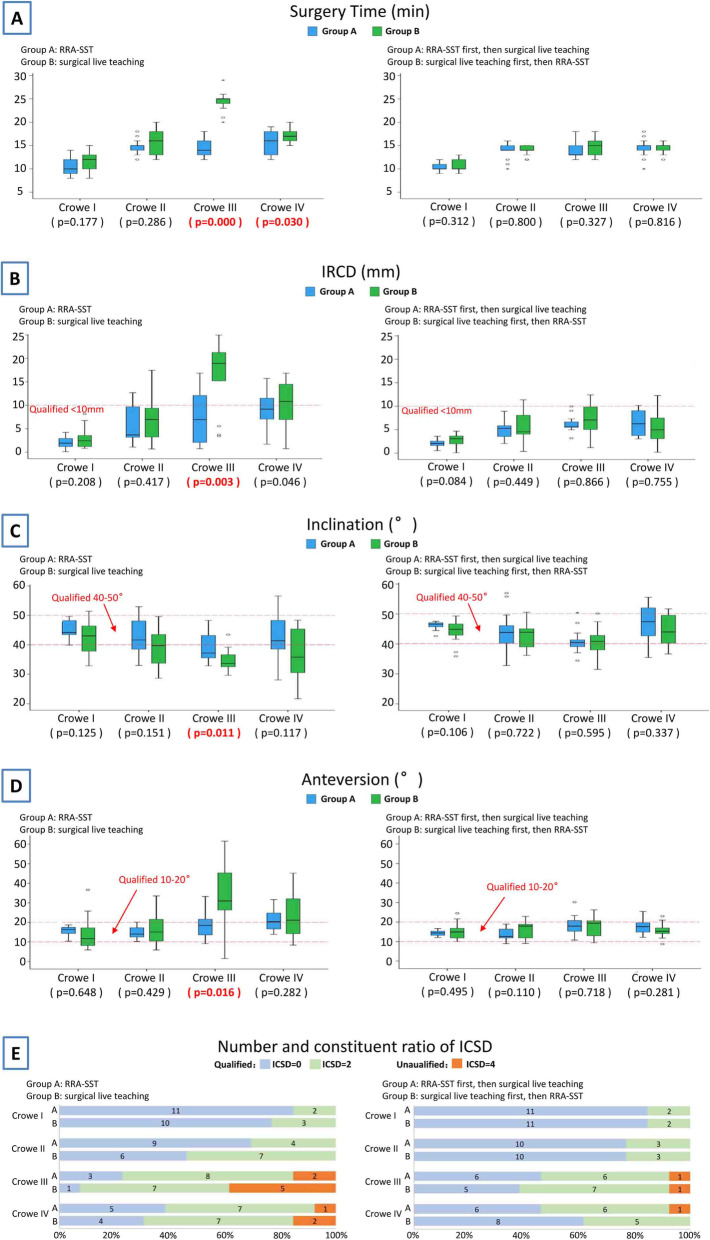
Objective test results: **a** Surgery time: when performed as the first test, RRA-SST significantly shortened the surgery times for Crowe’s classifications III and IV; **b** Ideal rotator center discrepancy (IRCD): when performed as the first test, RRA-SST produced accurate IRCDs for Crowe’s classification III; **c** Inclination: when performed as the first test, the inclination of Group A for Crowe’s classification III and Group B for Crowe’s classifications II, III, and IV were not within the qualifying range, but the differences in inclination for Groups A and B for Crowe’s classification III were still statistically significant; **d** Anteversion: when performed as the first test, RRA-SST can make anteversion for Crowe’s classification III significantly better; **e** Number and constituent ratio of the ideal cup size discrepancy (ICSD): when performed as the first test, RRA-SST could increase the number of perfect and qualifying ICSDs and reduce the number of unqualified ICSDs

**Fig. 5:**
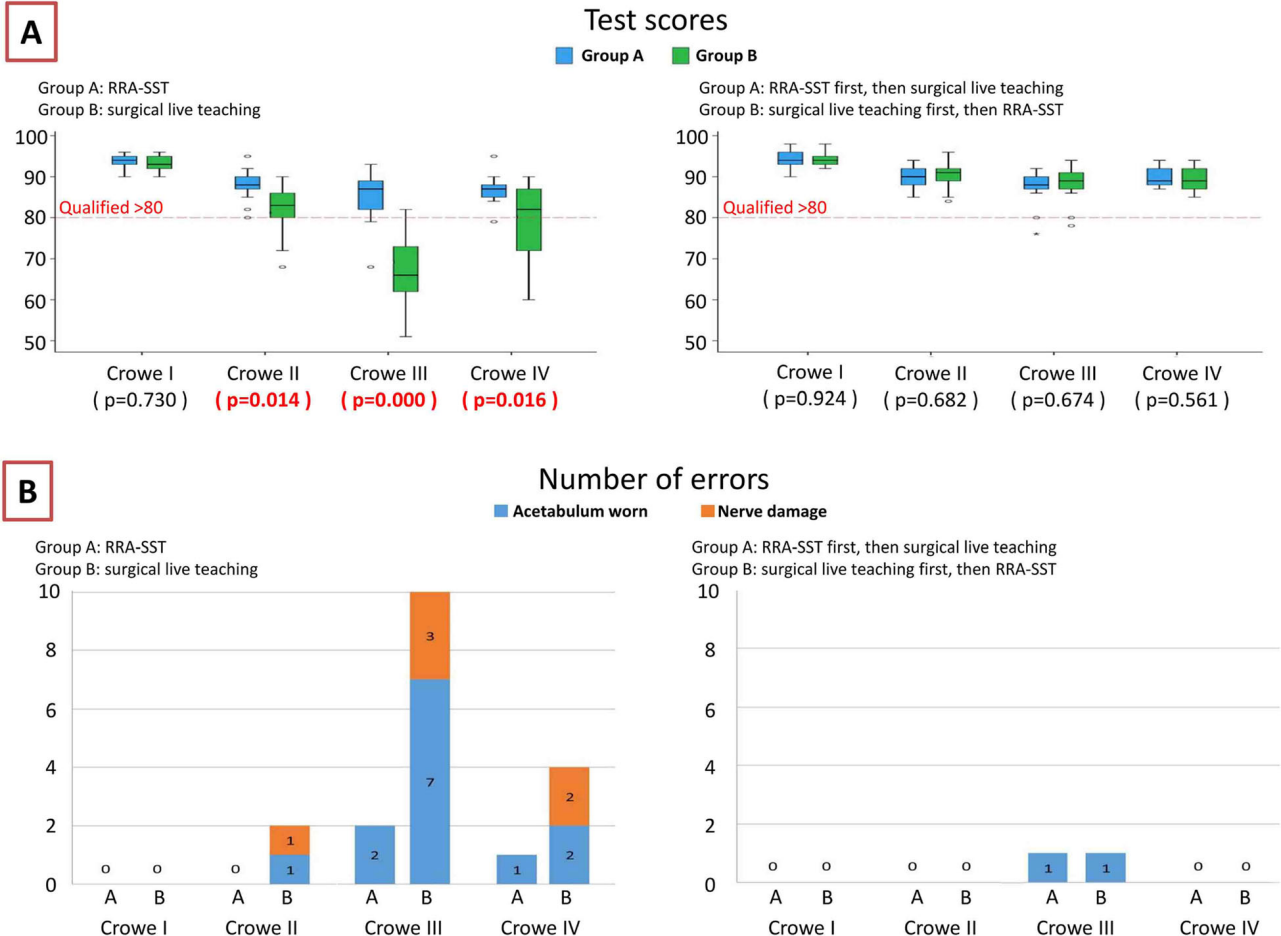
Subjective test results: **a** Test scores: when performed as the first test, the scores for Group B for Crowe’s classification III indicated poor performance, while the scores for Group B for Crowe’s classification IV indicated unqualified. RRA-SST can significantly improve the test scores for Crowe’s classifications II, III, and IV; **b** Number of errors: when performed as the first test, Group A made three errors while Group B made 16 errors; when performed as the second test, the number of errors in both two groups was greatly reduced

1. Surgery time: After the first test, RRA-SST significantly shortened the surgery time for Crowe’s classifications III and IV (Crowe III *p* = 0.030). After the second test, the surgery times for Groups A and B were significantly different within each Crowe’s classification type. However, the surgery time for Group B for Crowe’s classifications III and IV decreased significantly with RRA-SST, especially for Crowe’s classification III. The surgery times for Crowe’s classifications ranged from 24.54 ± 2.26 min to 14.77 ± 2.04 min (Fig. 4a).
2. IRCD: The qualifying time of IRCD was less than 10 mm. After the first test, we found the IRCD of Group B for Crowe’s classification III was far from the qualifying time (16.38 ± 7.50 mm), while the IRCD for Group B for Crowe’s classification IV was slightly unqualifying (10.44 ± 4.73 mm). After the first test, we also found that RRA-SST can make the IRCD for Crowe’s classification III significantly progressed (*p* = 0.003). However, after the second test, we found the IRCD of Group A did not improve significantly with live surgery training. The IRCD of Group B for Crowe’s classification III improved significantly with RRA-SST (7.14 ± 3.49 mml; Fig. 4b).
3. Inclination: The qualifying range of inclination was 40°–50°. After the first test, we determined the inclination for Group A for Crowe’s classification III (39.82 ± 5.35°) and Group B for Crowe’s classification II (38.99 ± 6.52°). Crowe’s classification III (34.78 ± 3.70°) and Crowe’s classification IV (37.38 ± 8.77°) were not within the qualifying range. However, after the first test, the differences in inclination for Groups A and B for Crowe’s classification III was still statistically significant (*p* = 0.011). After the second test, we found the inclinations for Groups A and B could be improved to the qualifying range with RRA-SST (Fig. 4c).
4. Anteversion: The qualifying range for anteversion was 10°–20°. After the first test, we found the anteversion for Group B for Crowe’s classification III was far from the qualifying range (33.34 ± 17.69°), while the anteversion for both groups for Crowe’s classification IV was slightly unqualified (20.43 ± 5.28° and 24.58 ± 12.37°, respectively). After the first test, we also found the RRA-SST can make anteversion for Crowe’s classification III significantly progressed (*p* = 0.016). After the second test, we did not find anteversion for all of Group A was significantly different with live surgery training. The anteversion of Group B for Crowe’s classifications III and IV improved significantly with RRA-SST (17.42 ± 5.52° and 15.88 ± 3.89°, respectively; Fig. 4d).
5. Number and constituent ratio of ICSD: The perfect ICSD size is zero, a qualifying size is less than or equal to two, and an unqualifying size is greater than two. After the first test, we found 10 unqualified ICSDs in both Groups A and B for Crowe’s classifications III and IV. RRA-SST, however, increased the number of perfect and qualifying ICSDs and reduced the number of unqualified ICSDs. After the second test, it was found that the number of unqualifying ICSDs decreased significantly in both groups (Fig. 4e).
6. Test scores: The qualifying range of test scores was greater than 80. After the first test, we found the scores of group B for Crowe’s classification III indicated poor performance (67.31 ± 8.77), while the scores for Group B for Crowe’s classification IV indicated the trainee was unqualified (78.92 ± 9.94). After the first test, we also found that RRA-SST can significantly improve the test scores for Crowe’s classifications II, III, and IV. The average test score for Group A for Crowe’s classification III was 85.15 ± 6.67 (*p* = 0.000). After the second test, we did not find the test scores of Group A were significantly different from that of each of Crowe’s classifications for the first test, but we found the test scores for Group B for Crowe’s classifications II, III, and IV improved significantly using RRA-SST compared with the first test (90.38 ± 3.52, 88.08 ± 4.55, and 89.23 ± 2.86; Fig. 5a).
7. Number of errors: After the first test, we found RRA-SST can significantly reduce the number of errors and the severity of errors. First, Group A made 3 errors while Group B made 16 errors. Second, Group B had 6 nerve injuries, but Group A had no such serious errors. After the second test, we found that the number of errors in both groups was greatly reduced to only one in each group. The two tests showed that treating Crowe’s classifications III and IV is still difficult for trainees (Fig. 5b).

### Subjective feedback

**Fig. 6:**
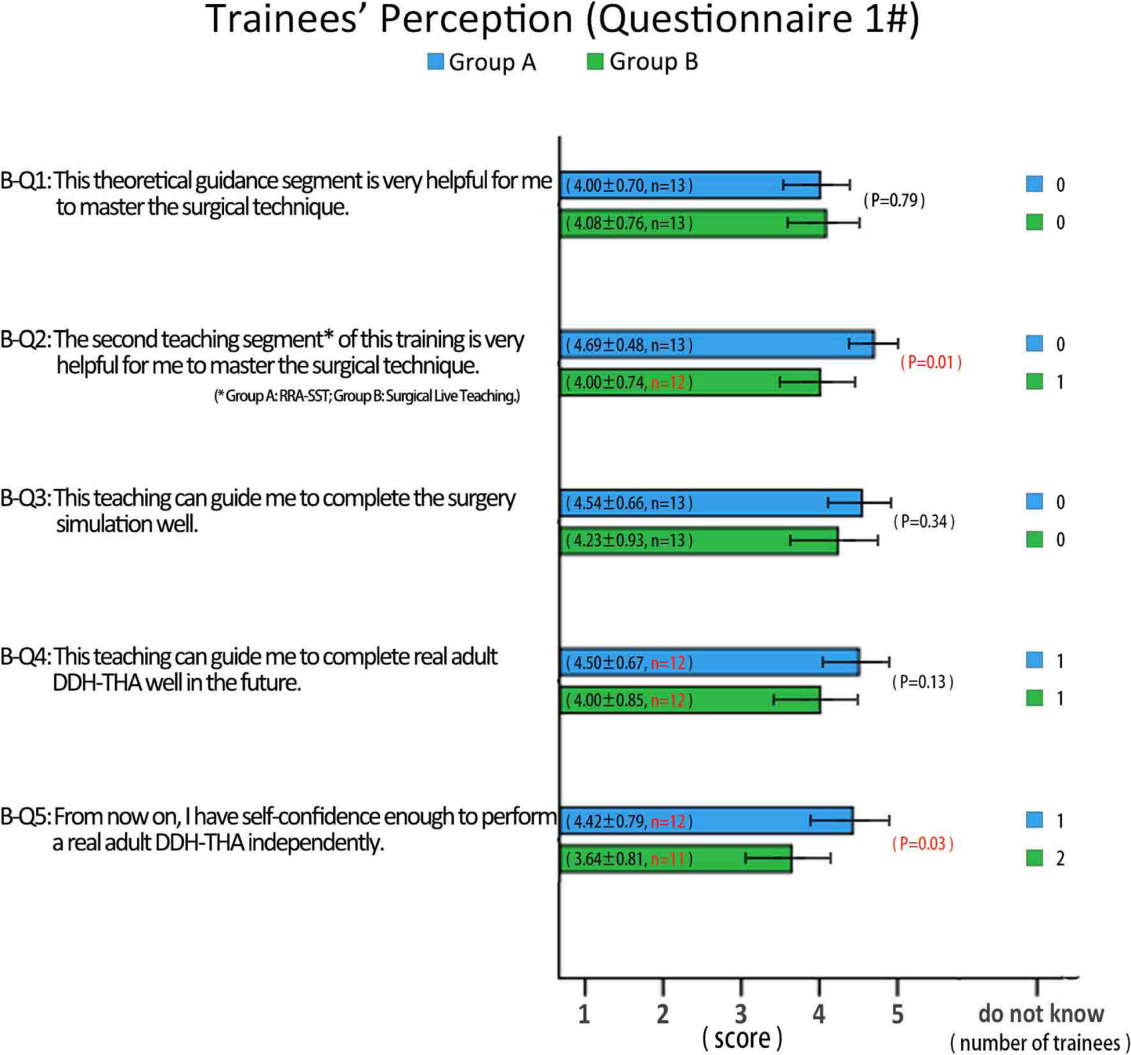
The feedback results for questionnaire #1, which was filled out after the first test

Questionnaire #1 was filled out after the first test and the results showed: 1) Both groups were satisfied with the teaching method they received, but evaluation of the training in RRA-SST for Group A was 4.69 ± 0.48, which was significantly higher than for Group B (4.00 ± 0.74, *p* = 0.01); 2) Both groups indicated the training they received with both the surgery simulation test and live surgery was helpful, but Group A gave a slightly more positive evaluation; 3) RRA-SST instills trainees with much more confidence than traditional surgical live teaching. After receiving RRA-SST, almost all the trainees in Group A were brave enough to carry out adult DDH-THA in their own practice, but the trainees in Group B showed little confidence (*p* = 0.03; Fig. 6).

**Fig. 7:**
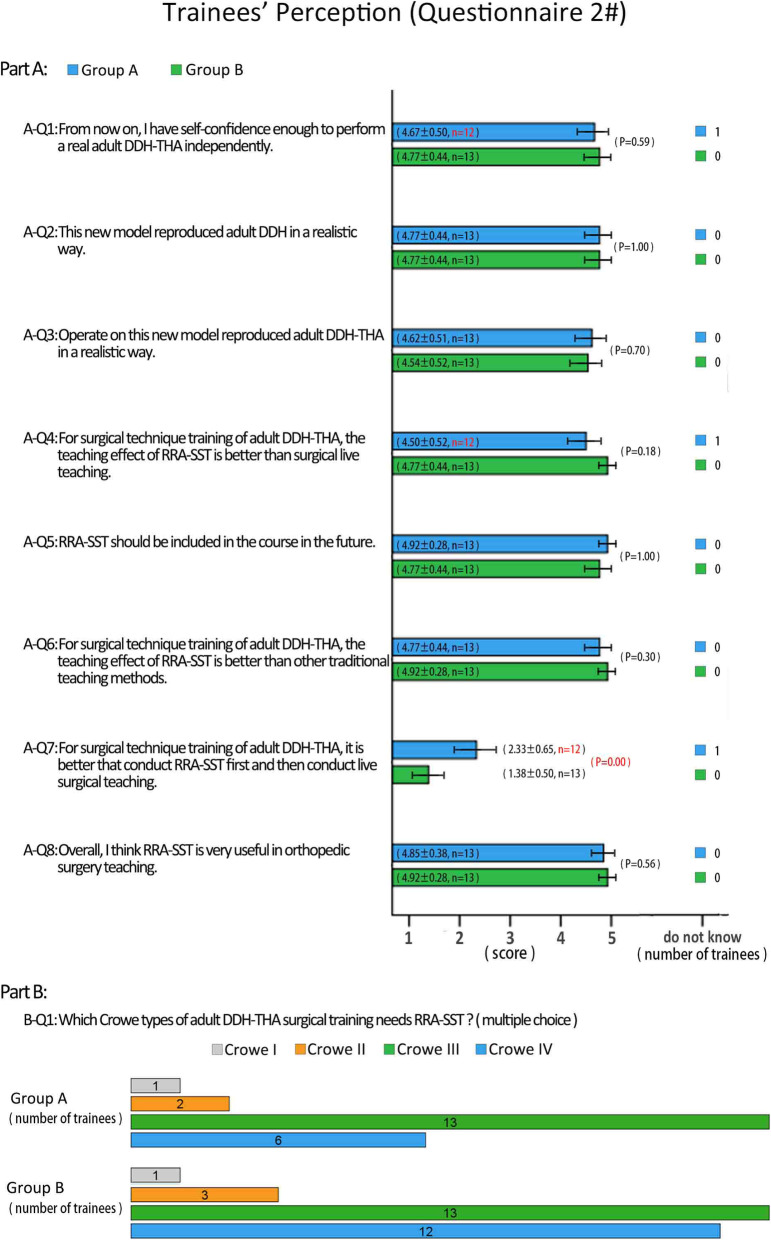
The feedback results for questionnaire #2, which was filled out after the second test

Questionnaire #2 was filled out after the second test and the results were as follows: 1) After complementary training with RRA-SST, Group B became as confident as Group A in carrying out adult DDH-THA in their own practice; 2) Both groups had positive attitudes towards this new teaching model, and also believed that it imitated the real state of adult DDH well and also imitated adult DDH-THA well; 3) Group A generally agreed that RRA-SST was better than surgical live teaching, but Group B strongly agreed that RRA-SST was better than surgical live teaching; 4) Both groups indicated it was better to conduct surgical live teaching first and then conduct RRA-SST, and Group B expressed this more firmly (*p* = 0.000). 5) Both groups strongly indicated that RRA-SST should be included in future training sessions and both groups indicated that RRA-SST is better than the traditional teaching method for adult DDH-THA. Both groups also suggested that RRA-SST would be very useful in orthopedic surgery training programs; and 6) Both groups suggested that Crowe’s classifications III and IV were most in need of RRA-SST. Crowe’s classification II was next in need for RRA-SST and both groups indicated that Crowe’s classification I did not have high need for RRA-SST (Fig. 7).

## Discussion

This study compared the training effects of RRA-SST with traditional surgical live training. The young doctor trainees who participated in the study were all orthopedic surgeons who were already in independent practice. The purpose of the training was to also master adult DDH-THA surgical skills. Before the study began, to reduce interference factors [26], we removed 10 trainees who did not meet the criteria of the study and we aimed to ensure consistency of trainee knowledge and experience levels. Consistency of trainers, teachers, and training locations was also maintained.

When analyzing the results of the first test, we found that the training effect of RRA-SST was better than that of traditional surgical live teaching. This finding was mainly true for Crowe’s classifications III and IV, and it was true secondarily for Crowe’s classification II. Specifically, RRA-SST can significantly shorten the surgery times of Crowe’s classifications III and IV, and can significantly improve the accuracy of the cup location (i.e., IRCD) and cup direction (inclination and anteversion) of Crowe’s classification III. RRA-SST can also significantly reduce the number of errors. In addition, from the perspective of the whole surgery testing process, RRA-SST can significantly improve the test scores in Crowe’s classifications II, III, and IV, which suggests the training effect of RRA-SST is better than traditional surgical live teaching. However, the accuracy of selection of the cup size (i.e., ICSD) when using RRA-SST is only slightly better than the accuracy in traditional surgical live teaching. In addition, we observed for Crowe’s classification I, the teaching effect differences between RRA-SST and traditional surgical live teaching were not obvious. The reason may be the operation steps and skills required for Crowe’s classification I adult DDH-THA were similar to an ordinary simple adult THA, and the trainees of the two groups had some surgery experience with simple adult THA.

The analysis of results for questionnaire #1 completed after the first test also supported the above results. The evaluations for training with RRA-SST showed higher scores than that of traditional surgical live teaching from multiple perspectives. In particular, the satisfaction with the RRA-SST teaching segment was significantly greater than that for traditional surgical live teaching. It is worth noting that participants who only received RRA-SST were able to carry out adult DDH-THAs in their own practice after training, but participants who only received the surgical live teaching were not able to do so.

Analysis of the results of the second test showed that the training for the two groups was considered to be at the same level. At this time, however, there was no difference between the teaching effects for Group A. whose members only received RRA-SST, and the teaching effects for both teaching methods. However, for Group B, especially for Crowe’s classifications III and IV, the teaching effects were greatly improved. After the RRA-SST teaching segment for Group B, the teaching effects for Group B were as good as or slightly better than for Group A. However, regarding the accuracy of selection of the cup size, the teaching effects for both methods after the second test were better than the effects after the first test. This difference should not be due to the teaching method, but due to the simulated surgery experience; that is, the advantage of experience accumulation was generated by observing multiple simulated surgeries.

The same results were found for questionnaire #2. The teaching effects and surgical confidence for Group B trainees achieved the level as that of Group A trainees. Almost all trainees indicated the RRA-SST method and the teaching aid model worked well. Moreover, most of the trainees considered the teaching effects with RRA-SST were better than the effects from the traditional teaching method. In addition, almost all trainees believed the RRA-SST should be included in the future, and they agreed with the point that RRA-SST is very useful in orthopedic surgery training. Although both groups of trainees indicated that it is better to conduct live surgical teaching first and then conduct RRA-SST training, Group B trainees strongly supported this view. The reason may be that Group B trainees experienced a strong difference in training effects through their own experience. In addition, both groups suggested that the types that needed RRA-SST most were Crowe’s classifications III and IV. Crowe’s classification II was second in need, and both groups indicated that Crowe’s classification I did not have a high need for RRA-SST.

By analyzing the results of the questionnaire, we found that some trainees were more optimistic about Questions B-5 of Questionnaire 1# than our team had expected. Although the test scores of the students who only received the traditional teaching method for Crowe’s classifications III and IV DDH-THAs indicated failure, they nevertheless indicated in the questionnaire they had the courage to carry out DDH-THA on their own. However, the morality of these students may not be considered a problem because most of them are still required to carry out DDH-THAs under the guidance of senior surgeons. Perhaps their intention when answering the questionnaire was to carry out DDH-THA for Crowe’s classifications I and II by themselves. Of note, through this study, we found that students performing DDH-THA for Crowe’s classifications I and II had a good command of both RRA-SST and traditional teaching methods.

Overall, RRA-SST can help the trainees who are already in practice and not experienced enough to master adult DDH-THA surgical techniques well. In addition, it can be considered that the teaching effects of RRA-SST are significantly better than traditional surgical live teaching for adult DDH-THA (Crowe’s classifications II, III, and IV). The reason may be that, for surgical technique teaching, the teaching method of live practical operations should have the best teaching effect, which is consistent with most other research on surgical technique teaching [27, 28]. However, it should be noted that it is perhaps unsurprising that Group B performed worse than Group A in their first simulation assessment because Group B was not familiar with the model. Moreover, we can reasonably predict that RRA-SST should also be helpful for other orthopedic surgical technique training. RRA-SST was based on a new teaching aid model, which had production costs and technical thresholds that were not high and can be reconstructed through typical actual cases realistically. What is more, these new teaching aid models are easier to obtain than cadaveric specimens. Therefore, this practical training based on the new teaching aid model should be promoted.

This study had some limitations. First, the sample size of this study was small. There were only 26 trainees in total in the two groups. Although some participants who may have caused interference were removed, it is difficult to completely avoid selection bias. Second, the questionnaires of this study were not able to eliminate the influence of the participants’ subjective preferences. For surgeons, it is likely to be subjective preference for practical operation live teaching [29]. Based on analyses of the training effects of the surgery simulation test, we concluded that RRA-SST improved the training effects. Third, this study only reflects the situation in China. In developed countries with more advanced medical education and a high level residential training system [30, 31], the significance of RRA-SST in teaching remains to be studied further.

## Conclusions

Our study showed that the use of RRA-SST improves trainee performance in a simulation assessment. RRA-SST can be helpful to trainees who are already in practice and not experienced enough to have mastered adult DDH-THA surgical techniques well. Perhaps RRA-SST will also be helpful in training programs for other orthopedic surgical techniques.

## Data Availability

The datasets used and/or analyzed during the study are available from the corresponding authors upon reasonable request.

## Abbreviations

RRA-SST: Surgery simulation teaching based on real reconstruction aid
DDH: Developmental dysplasia of the hip
THA: Total hip arthroplasty
IRCD: Ideal rotator center discrepancy
ICSD: Ideal cup size discrepancy
SM: Simulations and Models
